# An implementation strategy to improve the guideline adherence of insurance physicians: an experiment in a controlled setting

**DOI:** 10.1186/1748-5908-6-131

**Published:** 2011-12-21

**Authors:** Feico Zwerver, Antonius JM Schellart, Dirk L Knol, Johannes R Anema, Allard J van der Beek

**Affiliations:** 1 Department of Public and Occupational Health, EMGO Institute for Health and Care Research, VU University Medical Center, Amsterdam, The Netherlands; 2 Research Center for Insurance Medicine, collaboration between AMC-UMCG-UWV-VUmc, Amsterdam, The Netherlands; 3 Department of Epidemiology and Biostatistics, EMGO Institute for Health and Care Research, VU University Medical Center, Amsterdam, The Netherlands; 4 Dutch National Institute for Employee Benefits Schemes, Amsterdam, The Netherlands

## Abstract

**Background:**

The aim of this study was to investigate the efficacy of a newly developed implementation strategy for the insurance medicine guidelines for depression in the Netherlands. We hypothesized that an educational intervention would increase the insurance physicians' (IPs) guideline adherence in a controlled setting.

**Methods:**

Forty IPs were allocated in a randomised controlled trial (RCT) to an intervention group (IG) (n = 21) and a control group (CG) (n = 19). The IG received tailored training in applying the guidelines for depression, while the CG received an alternative programme. Baseline (T0) and follow-up (T1) measurements were conducted before and after the intervention within a period of two weeks. The intervention consisted of a workshop in which the evidence-based theory of the guidelines was translated for use in practice, with the help of various tools. The IPs had to write a case-report on the basis of video cases, two before and two after the training. Specially trained and blinded test IPs judged the case reports independently on the basis of six performance indicators. Primary outcome measure in the controlled setting of the trial was guideline adherence measured by six performance indicators on a scale of one to seven. Secondary outcome measure was knowledge of the guidelines for depression. Analyses were performed using Linear Mixed Models, and ANCOVA.

**Results:**

We found significantly higher scores in the IG than in the CG at T1 for both outcomes. The interaction effect (standard error; p-value) of group crossed with time was 0.97 (0.19; p < 0.0005) for guideline adherence in the controlled setting. The group effect at T1 for the knowledge test was 0.86 (0.40; p = 0.038).

**Conclusions:**

The newly developed implementation strategy for the insurance medicine guidelines for depression improved the guideline adherence of the trained IPs in disability assessments of clients with depression when performed in a controlled setting. Furthermore, the trained IPs showed gains in knowledge of the guidelines for depression.

**Trial registration:**

Netherlands' Trial Register NTR1863.

## Background

The implementation of guidelines, together with the difficulties and barriers that might occur when evidence-based medicine has to be translated into a physician's daily practice, has been the subject of various studies [1-4]. Results have suggested a sizeable gap between the ideal and the actual performance of physicians in the application of guidelines [5], and explanations for this gap have emerged. In existing studies, barriers to effective guideline implementation have been analysed at the level of the patient, the physician, and the healthcare organization [1,3,4]. In this study, we put the focus on the physician's guideline adherence. Our aim is to investigate in a controlled setting the influence of a newly developed implementation strategy on the adherence of the insurance physician (IP) to the depression guidelines. IPs evaluate the disability claims of employees, which is of great societal and financial importance; they write their assessments in a medical work disability report and the benefit level of the employee is defined on the basis of that report. We chose to study the insurance medicine guidelines for depression because depression accounts for a substantial and increasing proportion of long-term disability claims; this corresponds with worldwide trends that show that depression is expected to be one of the leading causes of disability-adjusted life years in 2020 [6,7].

We questioned whether the usual implementation of the guidelines for depression could be improved. After research, in which we used the Intervention Mapping method, we developed a newly implementation strategy for these guidelines [8]. It was found that this implementation strategy should include an interactive educational training and tools for IPs, with the aim to teach and facilitate IPs to apply these guidelines [8].

This aim is challenging because it means overcoming potential barriers such as negative aspects of physicians' behaviour in the adoption of guidelines. One of the summary points mentioned in an overview of reviews concerning the gap between research and practice is that passive dissemination of guidelines is generally ineffective [9,10]. Nevertheless, change in the behaviour of physicians was found for certain educational interventions, while in other more formal types of medical education there was no evidence of change [10]; interactive sessions that enhance participant activity and provide the opportunity to practice skills for instance were found to result in changes in the physician's performance [11]. In the development of our strategy, we took these findings into consideration by consulting educational experts and by assessing the needs of the IPs [8]. The IPs wanted tools such as a checklist or a guideline summary card to facilitate the use of guidelines in practice. The experts expected a multifaceted strategy with interactive educational sessions and the practice of skills to be most effective. After taking these findings into account, we developed an implementation strategy and evaluated its efficacy by setting up an randomised controlled trial (RCT) in which we compared implementation of the guidelines using this strategy with the usual levels of implementation in the Netherlands [12,13]. Primary outcome measure was the guideline adherence of the IP in a controlled setting, leading to the research questions: Does training IPs in applying the guidelines for depression improve their guideline adherence in the work disability assessment of clients with depression in a controlled setting? Additionally, does the strategy improve their knowledge of the guidelines for depression?

The Medical Ethics Committee of VU University Medical Center approved the study design and the Netherlands Trial Registration accepted the RCT: NTR1863.

## Methods

### Design

To determine the efficacy of training IPs in applying the guidelines for depression, we conducted an RCT in which we compared the intervention implementation strategy with the usual methods of training IPs by measuring their performances in disability assessments of clients with depression. The intervention was a training programme designed for IPs in which they learned to apply the guidelines for depression. This programme, together with the baseline and follow-up measurements, was integrated into a four-day postgraduate course located at the Netherlands School of Public and Occupational Health (NSPOH). At the NSPOH, we created a controlled setting in which we carried out the RCT. While the intervention group (IG) was trained in applying the guidelines for depression, the control group (CG) received an alternative programme of training in motivational interviewing that did not conflict with the intervention programme. The RCT took three days within a period of two weeks in March 2009. After the RCT had been ended, for reasons of recruitment and equal treatment for both groups, the CG received the same training as the IG, while the IG received the alternative programme; this was planned as the fourth day of the course, which was held three months later at the end of June 2009. At the NSPOH, we managed to create a laboratorial setting where we could measure each IP's work disability assessments of clients with depression played by actors on video. The training programme was developed for use in practice.

### Study population and recruitment procedure

In January 2009, IPs working at the Dutch National Institute for Employee Benefits (the Institute) were invited to attend a postgraduate course in applying disability assessments of clients with depression in the period from March to July 2009. The Institute is responsible for evaluating disability claims of employees. The inclusion criteria were being registered as an IP or still following the post-academic colloquium on insurance medicine and conducting disability assessments of clients as commissioned by the Institute. Participation was voluntary. The NSPOH was responsible for enrolling participants in the course during the period January to March 2009. Written informed consent was obtained from the participants of this study, when they entered the course.

### Allocation and blinding

The IPs who participated in the postgraduate education programme were individually allocated in order of registration to the IG or to the CG by means of a random-sequence table. To prevent an unequal allocation across both groups, the participants were stratified before randomisation on three prognostic factors: age, gender, and registration as an IP. The randomisation and stratification procedure was executed by a research assistant. After the stratification and randomisation procedure, the dates for the RCT were planned in cooperation with the NSPOH. Participants were assigned to either the IG or the CG by the research assistant, while the assignment was communicated to the participants by the NSPOH. Participants who were not available on the planned dates were excluded from the trial. The participants were blinded for the complete trial, including baseline, intervention or usual implementation programme, and follow up. The participants were informed about the fact that the course was part of a research project, but they were not informed about the design and were blinded for the type of group they participated in. Contamination between groups was not possible due to the design of the trial. The researchers were blinded for the collection of data. The data were coded by an independent research assistant.

### Measurements and data collection

Data were collected at the location of the NSPOH where the course took place. At baseline and at follow up, each IP assessed the disability of two clients, played by actors, who were presented separately on video. The actors played clients with depression that were reconstructed after real cases. The actors played their roles on the basis of extensive scripts, with room left for improvisation. Thereby, the actors realistically represented actual clients with depression. The videos showed the disability assessment encounter of a client and an independent IP (not a participant in the RCT), who was briefed to perform the assessment in complete accordance with the guidelines for depression. The decision phase of the assessment was not shown on the video. The IPs wrote their medical work disability reports immediately after watching each client on the video. All the medical work disability reports were collected during the RCT. Furthermore, each IP's knowledge of the guidelines for depression was measured at the start and at the end of the intervention and the control programme with the same set of knowledge questions.

### Outcomes

The primary outcome was the guideline adherence of IPs in the four work disability reports of clients with depression in the controlled setting of the RCT. We used performance indicators (PIs) to measure guideline adherence. The guideline adherence in these medical work disability reports is a proxy for the quality of the work disability assessments carried out by the IPs, given the guidelines for depression. The guideline adherence was measured by the six PI scale scores (range: 1 to 7). The six PIs were made up in the form of different decision trees with logic branches coming to an end with a score of not adequate (NA) or adequate (A). The main elements of the guidelines for depression, which should be detectable in an IP's work disability report on a client with depression, were covered by these PIs. A detailed description of the development and the reliability of the PIs can be found elsewhere [14]. In Table 1, the subjects of the six PIs are summarized. Furthermore, as a form of sensitivity analysis, we analyzed the guideline adherence in a different way as well *i.e.*, by using PIs scored as a binary outcome NA or A.

**Table 1:** Subjects of performance indicators for the guidelines for depression

PI 1	Correct diagnosis • DSM-IV criteria for depressive disorder
PI 2	Determination of severity of the disorder • Source: medical examination or *e.g*., information of curative physician, HRSD • Relation between severity of the disorder and the limitations
PI 3	Origin, course and prognosis of the disorder • Risk factors for depressive disorder • Course of depressive disorder • Substantiated prognosis of depressive disorder
PI 4	Co-morbidity • Presence or absence of co-morbidity • Influence of co-morbidity on prognosis and limitations
PI 5	Evaluation of care and cure • Level of information about claimant and medical treatment • Action for required information if necessary • Reasons for stagnation in recovery of functioning • Medical treatment related to rehabilitation
PI 6	Assessment of work limitations • Work limitations related to the severity of depressive disorder • Work limitations substantiated to insurance medicine standards

Adapted from Schellart *et al. *2011 [2]

The secondary outcome was the IP's knowledge of the guidelines for depression, which was measured with a knowledge test. The knowledge test was developed on the basis of the guidelines for depression. This test was piloted by two independent IPs, who were researchers from other research groups and not involved in our project. In the final version, the test consisted of 10 propositions derived from the guidelines to be scored true or false. Sum scores were calculated immediately before and after the intervention or control programme.

### Judgement of the medical work disability reports with PIs by test IPs

After the RCT was completed, the medical work disability reports of all participants were measured with PIs at baseline and at follow up by three pairs of senior test IPs. These test IPs had received separate training in which they learned to measure medical work disability reports using the PIs. Each medical work disability report from each participant from the RCT was judged blind by one pair of test IPs, which produced the PI scores per report. In cases of disagreement in the pair of Test IPs about a certain PI score, a consensus procedure was followed, resulting in one PI score. We measured the guideline adherence by the scoring of the six PIs as a scale. Taking into account the distance between the NA scores and A scores within the PIs, and the differences across the PIs, the scores were recoded in the following way to form the scale: (NA1 = 1), (NA2 = 2), (NA3 = 3), (NA4 to NA7 = 4), (A1 = 5), (A2 = 6), (A3, A4 = 7). A more extensive explanation of the scale and its reliability with two test IPs is published elsewhere [14].

### Intervention implementation strategy

The implementation strategy was developed on the basis of a needs assessment carried out by IPs and of semi-structured interviews held with psychiatrists, researchers, IP trainers with experience in post graduate education, and the psychiatrist who was member of the board that drew up the guidelines for depression. In this needs assessment, the needs of the IPs concerning the implementation of the guidelines were investigated. Their needs and the recommendations of the other experts were integrated into the intervention strategy using Intervention Mapping [11]. This intervention strategy consisted of a specific training programme for IPs in which they learn to apply the guidelines for assessing depression. Several evidence-based tools were developed to serve this goal intended to improve the applicability of the guidelines. Realistic cases of clients with depression presented at a video screening were used to enrich the training. Learning objectives for the IPs were: to use the tools for the improvement of their diagnostic skills, to improve their performances in the work disability assessment of clients with depression, and to write their findings and conclusions down in well-argued medical reports. The aim was to bring the IPs' reports more in concordance with the guidelines, *i.e.*, transparent, more evidence-based, and well-argued conclusions of clients' limitations in working ability. The participant's self-activation was stimulated in an interactive learning process with feedback given by the trainers. Two of the authors (FZ and JRA), both IPs themselves, were involved as trainers in the intervention programme. Appendix 1 gives an overview of the intervention programme.

### Sample size and measures

Sample size estimation was based on the minimum desirable change in the primary outcome that is the least sensitive, *i.e.*, the NA/A-score of the guideline adherence. For detecting a difference of 25% in the proportions of adequate scores between two independent groups, with a power of 80% and an alpha of 0.05 (two-sided), we needed 20 IPs in each arm of the RCT. Given the fact that each IP made two cases at baseline and at follow up and each case was scored with six PIs, each IP produced 12 NA/A-scores at both baseline and follow up.

### Statistical analysis

The baseline characteristics of the IPs in the two groups (CG and IG) were compared using crosstabs (Chi-square) for the categorical variables and independent T-test for the continuous variables.

Univariately, the outcome measures were analyzed with T-test for the scale scores of the PIs and the sum scores of the knowledge test. These tests were paired for the difference between T0 and T1 per group (CG or IG), and independently for the difference between groups (CG and IG) at T0 and T1. For the binary outcome measure, the differences between the groups (CG and IG) at the two time points (T0 and T1) were analyzed with crosstabs (Chi-square).

The scale outcome variable of the PIs (1 to 7) was analyzed using linear mixed models. Besides the effect of group, time, and their interaction effect on the outcome variable (the scaled PI score), we also corrected for the possible effects of the case, of the pair of test IPs, and of the kind of PIs per case on the PI scores of the participants. In our model, fixed effects were: intercept, group (CG, IG), time (T0, T1), pair of test IPs (1, 2, 3), group*time, case (B, C, D, E) within time, and PI (1 to 6) within case within time. Besides these fixed effects, a random coefficient for the intercept with as 'subject' the IPs (1 to 40) was calculated to account for possible clustering of the scores at IP level.

The binary outcome variable for the PI-score NA versus A was analyzed using Generalized Estimating Equations (GEE) with a logit link function, and with the same fixed effects as was the case in the linear mixed models analysis of the scale outcome variable, and for subject effect the IPs (1 to 40). Ancillary analyses were performed, using the differences in the estimated marginal means, to analyze the influence of case, pair of test IPs and PIs on the proportion of adequate scores.

For the knowledge test, the sum score of good answers per group before and after the training was calculated and analyzed using ANCOVA, with the sum score of the knowledge test at T1 as dependent variable and the sum score of the knowledge test at T0 and group as independent variables.

In the multivariate analyses possible confounding effects of background variables--of which the baseline characteristics of the IPs in the two groups (CG, IG) were significantly different--on the outcome variable were taken into account, *i.e.*, a change of the coefficient of the variable group of 10% or more. If so, a possible interaction effect of this confounding variable with the variable group on the outcome variable was analyzed as well. All analyses were performed using SPSS 15.02.

## Results

### Participant flow

**Figure 1:**
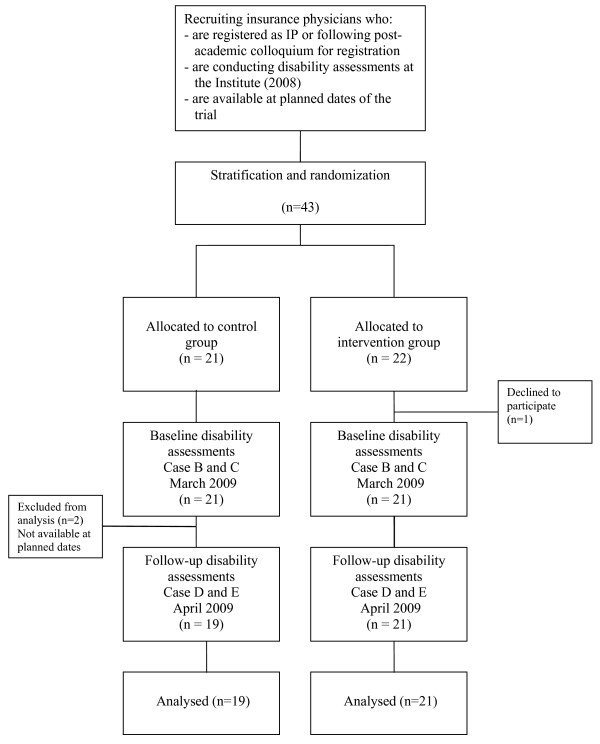
**Flow chart of the participants through the phases of the randomised controlled trial**. IP, Insurance physician. Institute, Dutch Institute for Employee Benefit Schemes.

Between January and March 2009 43 IPs applied for the course at the NSPOH. After the stratification and the randomisation procedure, 21 were allocated to the CG and 22 to the IG. One of the IPs who was allocated to the IG withdrew from the course and was lost to follow up. Two IPs who were originally allocated to the CG were not available on the planned dates for the CG; they participated for reasons of education in the IG but were excluded from the RCT. Therefore the CG consisted of 19 participants and the IG of 21 (See the flowchart in Figure 1). The 40 IPs completed the trial and all data were obtained, except for the knowledge test results of two IPs of the 19 IPs from the CG. These two IPs refrained from this part of the measurements for personal reasons.

### Baseline characteristics

Baseline characteristics revealed a significant imbalance between the CG and the IG only for the variable 'mean number of clients with depression assessed by an IP per month.' If necessary, the results of all the analyses were adjusted for this variable. The other baseline characteristics (*i.e.*, age, gender, being registered as an IP, *et al*.; see Table 2) were almost equally distributed across both groups (Chi-Square tests and T-tests were not significant). All participating IPs were currently conducting disability assessments at time of the RCT.

**Table 2:** Baseline characteristics of insurance physicians in control group (CG) and intervention group (IG)

Baseline characteristics	CG (n = 19)	IG (n = 21)
	Mean (sd) or percentage	
Age in years	50.5 (6.7)	51.1 (6.2)
Male	47%	52%
Weekly working hours	31.8 (9.9)	31.1 (9.2)
Years working as physician	21.7 (6.4)	23.5 (5.1)
Registered as insurance physician	84%	86%
Years working as insurance physician	15.4 (8.1)	15.6 (7.9)
Intensity of kind of professional activities	4.1 (0.8)	3.9 (0.8)
Number of clients with depression assessed per month*	9.3 (5.6)	5.3 (3.7)
Assessment time for depressed clients (minutes)	136.3 (62.3)	153.7 (48.4)
Assessments under the new disability act	68%	52%
Employee of the Institute	79%	81%
Attitude to guidelines in general (Scale 9 to 45)	30.8 (5.4)	31.7 (6.8)
Attitude to the GD (Scale 9 to 45)	31.8 (4.1)	33.9 (6.2)
Intention to use the GD (Scale 10 to 50)	34.5 (5.5)	35.0 (6.0)
Use (self-reported) of the GD (Scale 1 to 5)	3.1 (1.2)	3.0 (1.1)

* Significant difference between both groups, possible confounder

GD = Guidelines for Depression

Institute = the Dutch Institute for Employee Benefits Schemes

### Outcomes

In the controlled setting, the guideline adherence of the group trained IPs (n = 21) in the assessment of clients with depression (as measured with the scale outcome) increased by 16% compared with their guideline adherence at baseline, while the CG (n = 19) showed an 8% decrease in their guideline adherence at follow up. In the controlled setting, the guideline adherence of the trained IPs as measured with binominal outcome increased by 20% points to 71%, while the guideline adherence of the CG decreased by 5% points to 43%. The outcomes per different kinds of analysis are given in Tables 3, 4 and 5, and are illustrated below.

**Table 3:** Outcome measures control group (CG) and intervention group (IG) at T0 and at T1 and p-values between-group differences at T1

Guideline adherence IPs to GD	CG (19 physicians)		IG (21 physicians)		IG-CG Difference	
	
	T0	T1	T0	T1	T1	
	
	228 scores		252 scores			p-value
- Mean (SD) PI-sumscores (1-7)	3.6 (1.9)	3.3 (1.9)	3.8 (1.9)	4.4 (1.6)	T-Test	p < 0.0005

- Adequate scores (%)	48%	43%	51%	71%	Crosstabs	p < 0.0005

	CG (17 physicians)		IG (21 physicians)		IG-CG Difference	

**Knowledge test IPs on GD**	T0	T1	T0	T1	T1	

- Mean (SD) test scores (0-10)	5.1 (1.2)	5.1 (1.3)	5.5 (1.4)	6.3 (1.2)	T-Test	p = 0.006

**Table 4:** Results of the Mixed Models analysis of the primary outcome guideline adherence in a controlled setting.

	Group	Estimated means		Interaction effect (se) p-value
			
		T0	T1	
**PI-scale score** (1 to 7)	**CG**	3.62	3.32	0.97 (0.19) p < 0.0005
		
	**IG**	3.77	4.44	

Difference of Performance Indicator (PI) scale score outcomes between the insurance physicians (IP) in the Intervention group (IG) and the Control group (CG) at time T0 and T1. The estimated means and the interaction effect (time*group) with standard error (se) are presented.

Mixed Models Analyses: adjusted for fixed effects of Pair of test-IPs (1, 2, 3), Case (B, C, D, E) within Time, PI (1...6) within Case within Time and possible influences of clustering on the level of insurance physicians.

**Table 5:** Results of the Ancova analysis of secondary outcome knowledge

	Group	Estimated means	
		
		**T1**^**(1)**^	**T1**^**(2)**^
**Knowledge Test Sum Score **(0 to 10)	**CC**	5.24	5.29
	
	**IG**	6.19	6.15

**Group effect (se)** **p-value**		0.95 (0.36) 0.012	0.86 (0.40) 0.038

Difference of Knowledge Test Sum Score outcomes between intervention group (IG) and control group (CG) at T1. The estimated means, and the Group effect with standard error are presented.

^(1) ^Adjusted: covariate 'Knowledge Test Sum Score at T0' was evaluated at the mean value.

^(2) ^Adjusted: covariate 'Knowledge Test Sum Score at T0' and 'Mean Number of Clients with Depression Assessed by an IP per Month' were evaluated at their mean values.

The bivariate analyses of the data showed significantly higher scores for the IG at T1. The paired T-Test of the PI score between T0 and T1 was not significant for the CG (p = 0.092), but was significant for the IG (p < 0.0005). The unpaired T-Test of the difference between CG and the IG was not significant at T0 (p = 0.32) and significant at T1 (p < 0.0005). The crosstabs revealed similar results for the (percentages of) Adequate scores (See Table 3).

The unpaired T-test of the second outcome, the knowledge of the IPs of the guidelines for depression, showed no significant differences between groups at baseline (p = 0.28) and was significant at follow up (p = 0.006). The paired T-test between baseline and follow up was not significant for the CG (p = 0.84) and significant for the IG (p = 0.017)

The multivariate analyses of the data resulted in statistically significant differences between groups for the IPs' performances on applying the guidelines for depression. The mixed models analysis produced a significantly higher score at the PI-scale score, *i.e.*, guideline adherence, for the IG compared with the CG, accounted for possible effects of variables at different levels such as the pair of test IPs, the case or a certain PI used within a case (see Table 4). The GEE analysis, which also accounted for the effect of the same variables at the various levels, showed similar results for the binomial PI score (results not shown here). The estimated marginal means of the guideline adherence (NA/A score) per PI and per case showed that the trained IPs (IG) performed significantly better at each PI and at both cases at follow up (results not shown here).

The ANCOVA analysis of the secondary outcome knowledge test sum score produced significantly higher scores for the IG compared with the CG. The results of the ANCOVA analysis had to be adjusted for the confounding variable 'mean number of clients with depression, assessed by an IP per month' (see Table 5).

## Discussion

### Main findings and interpretation

Due to the newly developed implementation strategy, gains in guideline adherence and knowledge were obtained in the controlled setting of this study. The results of the knowledge test between the groups at follow up were significant, although the difference was smaller than that for guideline adherence.

In the controlled setting of this study, trained IPs performed their work disability assessments more in concordance with the guidelines than did those from the CG. It appeared that the trained IPs produced significantly more adequate scores in all six PIs. With these scores they distinguished themselves from the CG in each of the main points of the guidelines, as measured by the PIs at follow up.

After adjustment for confounding, the result of the knowledge test showed a smaller positive effect for intervention. A logical explanation for this is that fewer gains in knowledge of the guidelines can be achieved, due to the intervention, by IPs who are already more familiar with the disorder, suggesting that the greater the number of clients with depression an IP assesses per month, the more their knowledge of this disorder increases. Given the outcomes of the CG, it should be remarked that we found no indications for a learning effect from the measurements (assessing the work disability of the clients), because the guideline adherence in the CG seemed to decrease slightly in the follow-up measurement.

The scores of correct answers on the knowledge test were rather low for both groups at baseline and at follow up, but increased significantly for the IG compared with the CG at follow up. However, having knowledge of the guidelines does not imply application of the guidelines in practice. Guideline adherence as a concept should be regarded as behaviour involving more than knowledge. Furthermore, the main goal of training was that the IPs practised their skills in applying the guidelines, and not specifically that they improved their knowledge; the improvement in knowledge can be considered as a desirable side effect of the training.

### Strengths and limitations

The strength of this study was that the intervention implementation strategy was developed on the basis of the needs of IPs and the opinions of experts [11]. Another strong point was that we were able to measure quality with a valid and reliable instrument, the developed PIs [14]. In the analyses, we accounted for the possible effects of factors at different levels on the outcomes, such as the influence of the case, the pair of test IPs, and the PI itself. The results were confirmed in each of the different types of analysis. The design of this RCT, in which four different clients were each simultaneously assessed by groups of IPs, provided results that allowed us to draw sound and specific conclusions concerning the efficacy of the intervention. The developed implementation strategy improved guideline adherence in a controlled setting.

However, this design had limitations as well. We could not evaluate the effectiveness of the implementation strategy in practice, and thus the results of this study cannot be directly translated into practice. For practical reasons, we had to conduct the RCT in a fixed laboratorial setting. In this RCT, the disability assessments were performed under specific conditions: the clients were presented on a video screen, the participating IPs could not speak to them but had to assess their disability based on the information presented by the actor playing the client on the screen. Nevertheless, this RCT was based on and translated from the situation of IPs working in practice, and thereby offered us the optimal conditions for studying the efficacy of our newly developed implementation strategy. Another limitation of this study is the short time line of the RCT. This RCT contained one follow-up measurement, so that long-term effects of the training could not be evaluated; however, the justification for this short time line in the design of the RCT was the risk of contamination between groups *i.e.*, the possibility that the CG may be contaminated by the IG in the period after the IG had received the intervention programme. We therefore planned the intervention programme immediately after the follow-up measurement of the CG, making contamination of the CG by the IG impossible. The overall time interval between the start and the end of the RCT was no more than two weeks. By shortening the timeline of the trial, we limited the risk of influences from outside the laboratorial setting. Furthermore, selection bias of the IPs who participated in this study is possible since they participated voluntarily.

### Comparison with literature

The effectiveness of continuing medical education with the aim of stimulating physicians' guideline adherence has been evaluated for clinical care, primary care, and occupational healthcare [5,16-19]. The resulting overview was that most effects could be expected from multifaceted interventions characterized by mixed educational programmes with an active role for the physicians. Although in this RCT, where guideline adherence increased from 51% to 71%, this expectation was confirmed for the field of insurance medicine, our implementation strategy still has to be evaluated in practice where there may be more barriers to implementation than the physician's behaviour. Two primary care studies concerning multifaceted interventions in the implementation of guidelines did not demonstrate any increase in guideline adherence in practice [18,20]. In another study in primary care, multifaceted intervention strategy only modestly improved implementation of guidelines for low back pain [21]. The overall adherence rate to 70 guidelines in primary care within a period of 10 years was 67% [2]. For mental health disorders the figures of guideline adherence were even lower. In occupational healthcare, guideline adherence of 39% was found for mental health problems [22], while in primary care guideline adherences for depression and for anxiety disorders of 42% and 27%, respectively, were reported in a cross-sectional study [23]. Our findings showed that with a multifaceted strategy for a mental health disorder such as depression sizeable gains in guideline adherence could be achieved in a controlled setting. We can add to the conclusions of another insurance medicine study [24], in which it was found that a workshop improved evidence-based skills and self-efficacy, that an evidence-based medicine approach can be successfully adapted to the field of insurance medicine.

### Practice implications

The educational intervention was evaluated in this study with the participation of a limited group of IPs. Now that the efficacy of this training has been shown in the controlled setting of this study, distribution to other IPs is recommended. Though this training was developed for the guidelines for depression, with adjustments it could be used for the implementation of other insurance medicine guidelines as well. The results of the developed implementation strategy did not show evident barriers at the level of the physician. Potential organizational barriers, such as available time for a physician to apply guidelines in practice, could not be investigated in this RCT. However, this training programme can be seen to suit the needs of physicians and their employers, because its implementation requires the investment of only one day of the physician's time, while the return proves to be relatively high. The RCT was carried out in a controlled setting for practical reasons (*i.e.*, to have groups of IPs assessing the same videotaped clients simultaneously), but the implementation strategy (the training programme with evidence-based tools) itself is ready to be carried out in the real world setting. Finally, clients are expected to benefit from 'guideline proof' assessments, because the quality of these assessments is higher.

## Conclusions

In this study, the efficacy of a newly developed implementation strategy for the insurance medicine guidelines for depression was evaluated in an RCT. In a controlled setting, the implementation strategy did improve the guideline adherence of IPs in the disability assessments of clients with depression and gains in knowledge concerning the guidelines were achieved. Though the guideline adherence of the trained IPs increased sizeably under the specific conditions in this study, it is yet not known whether these effects will be retained in the long term. Therefore, further research on the long-term effectiveness of this educational intervention is needed. This educational intervention is suitable for practice, because it combines a high success rate with low investment as the training takes only one day of the physician's time.

## Competing interests

The authors declare that they have no competing interests.

## Authors' contributions

The authors declare that they participated in the study and made the following contributions to the study. AJMS, FZ, JRA, DLK, and AJvdB contributed to the conception and design of this study. AJMS, FZ, and DLK contributed to the analysis. FZ and AJMS wrote the manuscript. JRA, DLK, and AJvdB revised and commented on the manuscript. AJMS, JRA, and AJvdB will act as guarantors of this study. AJvB had full access to all data in the study and had final responsibility for the decision to submit for publication. All authors read and approved the final manuscript.

## Appendix 1. Intervention implementation

Intervention goals

To improve the IP's knowledge and skills and self-efficacy in applying the guidelines for depression. Practice reinforcement of the IP's assessments of clients with depression.

Learning objectives for the participating IPs

• Learn to perform disability assessment in concordance with guidelines for depression.

• Learn to apply tools to recognize depression and to assess the working ability of claimants with depression.

• Learn to base conclusions in the disability assessments on convincing arguments.

• Learn to write powerful, more transparent reports.

Intervention content

• Explanation of the evidence based content of the guidelines for depression by an IP trainer.

• Translation of the guidelines for depression by IP trainer into six main elements, which are relevant for use in daily practice of the IP: diagnostics DSM IV, severity of the disorder, course, risk factors, co-morbidity, judgement of treatment and therapy, assessment of work ability.

• Disability assessment of a client with depression presented on video.

• Writing assessment report on the client with depression.

Educational strategy

Course manual:

• Learn objectives, list of used literature, suggestions for further reading.

• The complete guidelines of depression in book format.

• Resume of the common principles of reasoning and in particular applied for the guidelines for depression (with practical examples).

• Tools with the aim of making the guidelines easier to use in practice: desk mat, summary with main elements from the guideline/evidence based medicine, checklists, Hamilton Depression Rating Scale [15]

• Handouts of presentations by psychiatrist and the trainer IP.

One-day training:

• Debriefing from the baseline measurements.

• Taking a knowledge test concerning the guidelines for depression.

• Inter-active presentation by the trainer IP concerning the guidelines for depression. The trainer translates the guidelines into the insurance physician's daily practice.

• Client with depression played by an actor is presented on video to the group.

• Workshop with IPs in subgroups learning to use practical tools for the assessment of the client with depression. Presentations of the findings per subgroup to the complete group, with feedback from the IP trainer. Modelling of written arguments in the assessment reports about the client with depression by the trainer IP.

• Evaluation of the training and taking the same knowledge test as at the start of the training.

IP-Insurance physician
